# Foraging areas, offshore habitat use, and colony overlap by incubating Leach’s storm-petrels *Oceanodroma leucorhoa* in the Northwest Atlantic

**DOI:** 10.1371/journal.pone.0194389

**Published:** 2018-05-09

**Authors:** April Hedd, Ingrid L. Pollet, Robert A. Mauck, Chantelle M. Burke, Mark L. Mallory, Laura A. McFarlane Tranquilla, William A. Montevecchi, Gregory J. Robertson, Robert A. Ronconi, Dave Shutler, Sabina I. Wilhelm, Neil M. Burgess

**Affiliations:** 1 Psychology Department, Memorial University, St. John’s, NL, Canada; 2 Department of Biology, Acadia University, Wolfville, NS, Canada; 3 Biology Department, Kenyon College, Gambier, OH, United States of America; 4 Wildlife Research Division, Environment and Climate Change Canada, Mount Pearl, NL, Canada; 5 Canadian Wildlife Service, Environment and Climate Change Canada, Mount Pearl, NL, Canada; 6 Ecotoxicology & Wildlife Health Division, Environment and Climate Change Canada, Mount Pearl, NL, Canada; Liverpool John Moores University, UNITED KINGDOM

## Abstract

Despite their importance in marine food webs, much has yet to be learned about the spatial ecology of small seabirds. This includes the Leach’s storm-petrel *Oceanodroma leucorhoa*, a species that is declining throughout its Northwest Atlantic breeding range. In 2013 and 2014, we used global location sensors to track foraging movements of incubating storm-petrels from 7 eastern Canadian breeding colonies. We determined and compared the foraging trip and at-sea habitat characteristics, analysed spatial overlap among colonies, and determined whether colony foraging ranges intersected with offshore oil and gas operations. Individuals tracked during the incubation period made 4.0 ± 1.4 day foraging trips, travelling to highly pelagic waters over and beyond continental slopes which ranged, on average, 400 to 830 km from colonies. Cumulative travel distances ranged from ~900 to 2,100 km among colonies. While colony size did not influence foraging trip characteristics or the size of areas used at sea, foraging distances tended to be shorter for individuals breeding at the southern end of the range. Core areas did not overlap considerably among colonies, and individuals from all sites except Kent Island in the Bay of Fundy foraged over waters with median depths > 1,950 m and average chlorophyll *a* concentrations ≤ 0.6 mg/m^3^. Sea surface temperatures within colony core areas varied considerably (11–23°C), coincident with the birds’ use of cold waters of the Labrador Current or warmer waters of the Gulf Stream Current. Offshore oil and gas operations intersected with the foraging ranges of 5 of 7 colonies. Three of these, including Baccalieu Island, Newfoundland, which supports the species’ largest population, have experienced substantial declines in the last few decades. Future work should prioritize modelling efforts to incorporate information on relative predation risk at colonies, spatially explicit risks at-sea on the breeding and wintering grounds, effects of climate and marine ecosystem change, as well as lethal and sub-lethal effects of environmental contaminants, to better understand drivers of Leach’s storm-petrel populations trends in Atlantic Canada.

## Introduction

Small (< ~150 g) procellariiform seabirds are important components of food webs in marine ecosystems worldwide. Yet, owing to their cryptic nature at colonies and at sea, their ecology is relatively poorly known. Historically they have been too small to track, so there is a particular knowledge gap around their spatial ecology, including movement patterns, migration routes, and wintering areas. This situation is being redressed with recent availability of miniaturized (~ 1 g) tracking devices, which can record movements of even the smallest species over extended periods of time [1–7].

**Fig 1:**
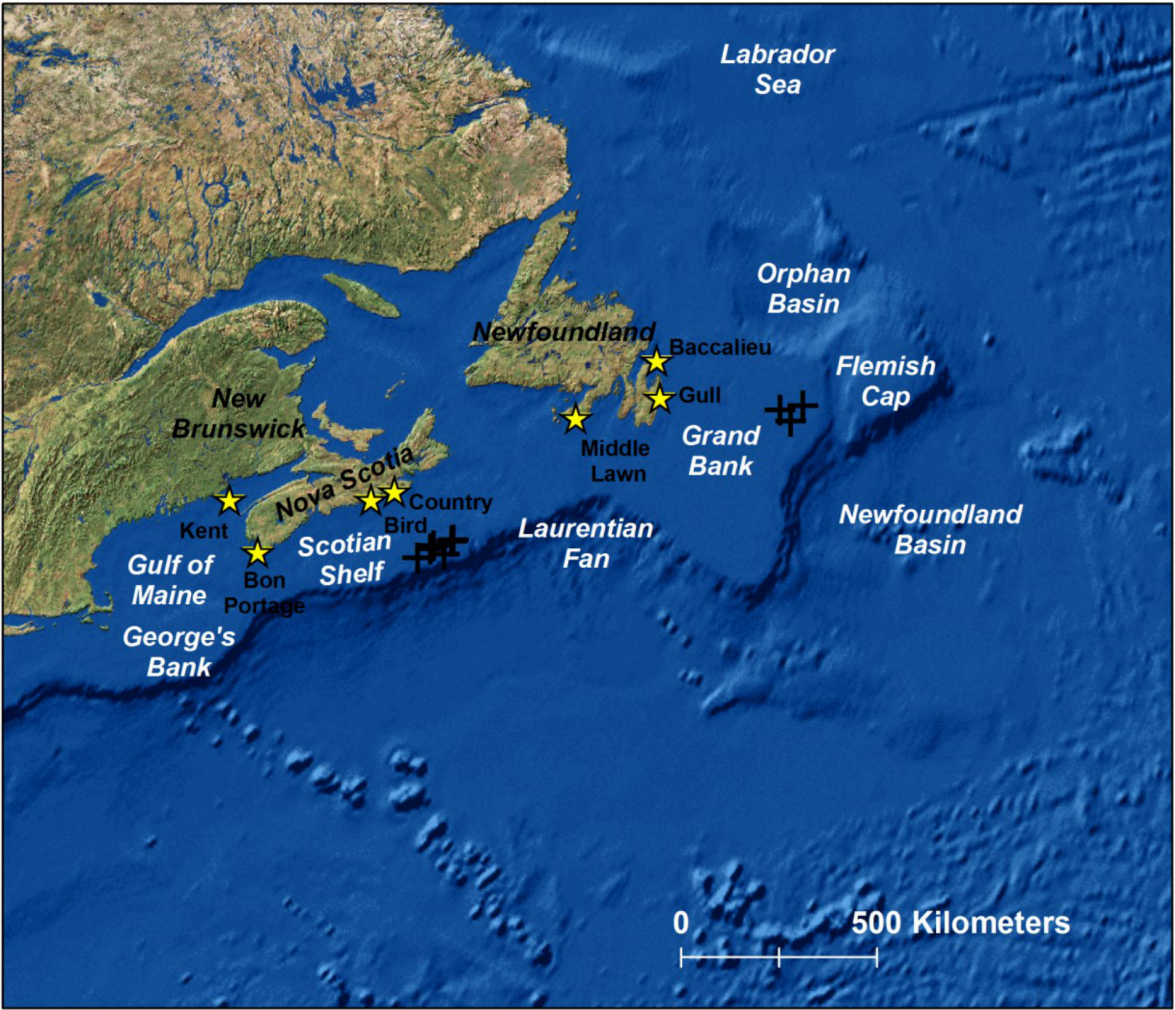
Study area. Eastern Canadian colonies from which Leach’s storm-petrels were tracked in 2013 and 2014 (yellow stars) along with oil and gas production platforms (**+**). Also indicated are place names mentioned in the text.

Leach’s storm-petrel *Oceanodroma leucorhoa*, a small (~45 g), abundant, burrow-nesting procellariiform seabird, breeds throughout the North Atlantic and North Pacific Oceans [8]. In the Northwest Atlantic, the bulk of the breeding population is situated off eastern Newfoundland and within the Saint-Pierre and Miquelon Archipelago off Newfoundland’s south coast [8,9] (Fig 1). In this region, Leach’s storm-petrels are highly pelagic during breeding, foraging over deep waters far from colonies to access mesopelagic lanternfish (Myctophidae), one of their preferred prey for provisioning chicks [10,11]. Recent tracking at 2 sites in Nova Scotia also indicates a reliance on pelagic habitats with birds ranging on average ~600–1,000 km and travelling cumulative distances of ~1,300–2,200 km from colonies on foraging trips during the incubation period [3].

Leach’s storm-petrels are *Vulnerable* on the IUCN Red List [12] and declining at many colonies in Atlantic Canada (Table 1) [13–17], including at Baccalieu Island, Newfoundland, which supports the species’ largest population [14]. Though cause(s) of these population declines are unknown, potential cumulative drivers include predation at colonies by large gulls (*Larus* spp.) [15,18,19] and mammals (e.g., meadow voles, *Microtus pennsylvanicus*, at Country Island, Nova Scotia)[3], high levels of contaminants in eggs and other tissues [20–23], threats associated with light pollution from vessels and offshore oil and gas platforms [24–26], and ongoing climate and marine ecosystem changes [27]. A lack of information on the spatio-temporal distribution of Leach’s storm-petrels has hampered our ability to assess some of the threats they face, because the influence of distribution on relative vulnerability to spatially explicit threats is not well understood. To help address the information gap, tracking studies were initiated at a network of colonies throughout the species’ eastern Canadian breeding range. During incubation at 7 study colonies, our objectives were to determine the: (1) characteristics of foraging trips, (2) location of foraging areas and their associated habitat characteristics, (3) extent of spatial overlap among colonies at sea, and (4) identity of colonies whose foraging ranges intersected with offshore oil and gas operations.

**Table 1:** Population size and trend, along with numbers of Leach’s storm-petrels *Oceanodroma leucorhoa* tracked using global location sensors (GLS) from 7 eastern Canadian breeding colonies during incubation in 2013 and 2014.

			Tracking			
Colony (latitude, longitude)	Breeding population size (pairs)	Population trend	Year	N GLS (deployed, birds retrieved, datasets)	N filtered locations	GLS Model (elevation angle)
Baccalieu Is., NL	1,976,665[13]	Declining[13,14]	2013	16, 6, 6	78	5040 (-3.5), 5440 (-3.5)
(48.12°N, -52.8°W)			2014	19, 15, 13	209	5440 (-3.5), 5540 (-4.0)
Gull Is., NL	179,743[13]	Declining[13,15]	2013	12, 8, 7	152	5040 (-3.5), 5440 (-3.5), 5540 (-4.0)
(47.27°N, -52.77°W)			2014	19, 17, 16	273	5440 (-3.5), 5540 (-4.0)
Middle Lawn Is., NL (46.87°N, -55.62°W)	10,791[13]	Declining[15]	2014	18, 9, 9	163	5440 (-3.5), 5540 (-4.0)
Country Is., NS (45.1°N, -61.54°W)	11,990[13]	Declining[13]	2013	15, 9, 9	148	5540 (-4.0), 5740 (-4.7)
Bird Is., NS	1,200[13]	Unknown	2013	15, 12, 11	192	5540 (-4.0), 5740 (-4.7)
(44.87°N, -62.28°W)						
Bon Portage Is., NS	50,000[16]	Unknown	2013	17, 14, 14	227	5540 (-4.0), 5740 (-4.7)
(43.46°N, -65.74°W)			2014	18, 16, 16	192	5540 (-4.0)
Kent Is., NB	25,000[13]	Unknown	2013	20, 18, 17	548	5540 (-4.0)
(44.58°N, -66.8°W)			2014	20, 16, 15	388	5540 (-4.0)
			**Total**	189, 140, 133	2,570	

## Materials and methods

### Ethics statement

Approval of the protocols used in this study was granted by the Animal Care Committee of Environment Canada (Wildlife and Landscape Science Directorate/Canadian Wildlife Service, Ontario; Protocol numbers 13GR01 and 14GR01). All necessary access permits were obtained from the Provincial Governments of Newfoundland and Labrador, Nova Scotia, and New Brunswick and all regulations were followed.

### Fieldwork

During 2013 and 2014, foraging movements of breeding Leach’s storm-petrels from 7 Northwest Atlantic colonies (Fig 1), spanning Baccalieu Island, Newfoundland to Kent Island, New Brunswick were studied using global location sensors (GLS) from the British Antarctic Survey (BAS, Cambridge, UK; model MK5740 [0.9 g, 21.9 × 7.9 × 3.8 mm, with a 6.8-mm light sensor stalk]) or BioTrack (Dorset, UK, models MK5040 [0.75 g, 20 × 8 × 3 mm], MK5440 [0.9 g, 22 × 8 × 3 mm, with 10-mm light sensor stalk], MK5540 [0.9 g, 22 × 8 × 3 mm]). With the exception of five MK5040 that were deployed on the leg, GLS were deployed on the back. Total mass of GLS plus attachment materials was ≤ 1.3 g or ~2.9% of average adult body mass during incubation (~45 g; [3]). Incubating birds were randomly selected for device attachment. At all sites except Kent Island, back-mounted GLS were deployed using subdermal sutures (Ethicon Prolene 4–0, FS-2, 19 mm or Ethicon Ethilon 5–0, PS-3, 19 mm) following procedures described in Pollet et al. [3,4]. Briefly, the skin was lifted to avoid muscle and two sutures were used, one between the scapulae and the other ~2 cm posterior to the scapulae, to secure the front and back ends of the device, respectively. Sutures were tied using 4 double square knots and a small bead of superglue was placed on the underside of the device and on the suture knots to enhance attachment. Suture attachment has no effect on mass of adults or reproductive success in Leach’s storm-petrels; however, chick growth for birds carrying sutured GLS was slower than for controls [3]. At Kent Island, devices were attached to the back using superglue and chiffon. In advance of deployment, a piece of chiffon matching the outline of the GLS was glued to the underside of the device. A matching patch of skin along the centreline of the back was prepared by snipping feathers and leaving 1–2 mm at the base of each feather shaft. The GLS was then glued to the remaining feather shafts that partially covered the skin. For the leg attachments, devices were mounted on two predrilled soft metal bird bands using monofilament, tied with a buntline hitch, and secured with superglue. Birds were returned to their burrows following device attachment, usually within 15 minutes. Across sites and years, most GLS were deployed and retrieved during the incubation period. Following typical ~2–4 week deployments, sutured and leg-mounted GLS were removed by cutting the sutures and monofilament, respectively. At Kent Island, GLS birds were recaptured and data were downloaded as they approached the end of the incubation period. These glued devices remained in place until attachments failed and they were subsequently lost.

### GLS processing, analysis and validation

GLS were equipped with an internal clock and battery and measured light levels every 1 or 2 minutes, logging maximum levels recorded at 2-minute intervals. Resulting GLS records (Table 1) were first decompressed using BASTrack software (Cambridge, UK) and subsequently processed in MultiTrace Geolocation (Jensen Software Systems, Laboe, Germany) using methods outlined in Phillips et al. [28]. Following application of this approach, bird locations were derived using a threshold method. Briefly, sunset and sunrise times are estimated in MultiTrace from thresholds in light curves; day/night length provides an estimate of latitude, and the timing of local midday/midnight, relative to GMT, provides an estimate of longitude. This procedure produces two locations per day, corresponding to local midday and midnight. Mean positional error ± SD of similar GLS devices deployed on free-ranging Black-browed albatrosses *Thalassarche melanophrys* has been estimated at 186 ± 114 km [28].

Data were processed in MultiTrace using a light level threshold of 10. Elevation angles for each GLS model were selected based upon information collected during a pre-deployment ground-truthing period, when GLS were placed at known locations for several days. Elevation angles producing the best fit with the ground-truth locations were chosen for models deployed within a single province (Table 1; the MK5040, MK5440, and MK5740). Following previous studies, a common light angle was applied for the MK5540 model, which was deployed at all colonies [29,30]. Because one aim of this study was to examine the extent to which foraging zones overlapped among colonies, this approach ensured that observed spatial patterns were not driven by differences in processing.

For each GLS record, all sunset/sunrise transitions were examined in MultiTrace and a comment was inserted if there was obvious light interference. Resulting locations were then individually examined in ArcGIS by the same observer (AH) and clearly erroneous locations were removed (e.g., those lying outside the breeding range, or those requiring unrealistic rates of movement) [28]. Spatial analyses were restricted to valid locations.

Direct examination of light records in MultiTrace Geolocation helped distinguish time at sea from time in the burrow, and subsequently the duration of individual foraging trips during the incubation period. Attendance of eggs in burrows resulted in complete darkness for up to 7 days during this phase. For each foraging trip, we estimated the duration (1-day resolution), approximate maximum foraging range (furthest distance from the colony), and the cumulative distance travelled (both great-circle, in km). Distance calculations were anchored by start and end points at the colony.

We assessed the suitability of using threshold derived GLS positions to characterize foraging movements of incubating storm-petrels in our region by making two comparisons. First, for a subset of the current data (specifically, 2013 data from Country and Bon Portage Islands), we compared threshold derived estimates of foraging parameters (i.e., foraging ranges and cumulative travel distances) with those previously derived using Bayesian techniques that control for error in location estimates [3]. In addition, our GLS estimates of foraging parameters for Gull Island in 2013–14 were compared with those from high-precision tracking at this site in 2016, when GPS loggers (Pathtrack Ltd., Otley, UK; model nanoFix-GEO mini [1.0 g, 20 × 10 × 4.5 mm, with a 5-cm antennae]), recording at 2-h intervals, were used to track the foraging movements of incubating birds.

The influence of colony of origin on the characteristics of Leach’s storm-petrel foraging trips was examined using general linear mixed effects models (LME) fit by restricted maximum likelihood. Mixed modeling was employed to account for the fact that data obtained from the same individual are likely correlated, so individual was set as a random effect. *F*-tests were used to assess the significance of effects, with follow-up tests conducted using the “effects” package in R [31], in which case groups were considered different if their 95% confidence intervals did not overlap. Because not all colonies were studied in both years, colony effects were examined separately in 2013 and 2014. Models were built and statistics were run using R software, and unless stated otherwise, values are presented as means ± SD.

Locations of birds at sea were mapped in ArcGIS 9.3 (ESRI, Redlands, CA, USA). Kernel contours describing the utilization distribution (UD) for each colony (pooled across years) were created using the ‘adehabitatHR’ package [32] in R (version 3.2.1) [31], with smoothing parameters (*h*) chosen via LSCV. For each colony, the contour (%UD) delineating the core area was defined objectively using the method of Vander Wal & Rodgers [33] and the 95% UD was used to delineate the peripheral or home range area [34,35]. Core areas were defined as the portion of the home range area where time spent (and, hence, intensity of use) was maximized relative to the periphery [33]. Spatial overlap of colony core and home range areas was determined by overlaying core and 95% UDs, respectively, and calculating their intersection (A_o_, km^2^). The percent of area shared by two colonies could range from 0–100%, and followed this equation [34,36]:

$${\%}_{\mathrm{SHARED}\;\mathrm{AREA}}{=[A}_{o}{]÷[(A}_{\mathrm{Colony}1}{-A}_{o}{)+(A}_{\mathrm{Colony}2}{-A}_{o}{)+A}_{o}]$$

For each colony, spatial overlap with offshore oil and gas platforms was assessed by determining whether platforms intersected the UD, and where intersection occurred, noting whether it was within the core or the periphery.

### Ocean habitat characteristics

Leach’s storm-petrel foraging habitat was characterized by using Marine Geospatial Ecology Tools (MGET; [37]) to sample bathymetry and remotely-sensed sea surface temperature (SST) and chlorophyll *a* concentration (chl *a*) at GLS locations within the core area of each colony. Bathymetry was determined from ETOPO2 grids, and SST and chl *a* data were monthly Aqua MODIS products mapped at 9-km resolution. Habitat characteristics within core areas were compared with values for the same variables extracted for ≥ 10,000 random points within each colony’s mean maximum foraging range (i.e., the potentially available habitat). For dynamic variables, random points were assigned a date corresponding to the midpoint of the tracking sessions, in relative proportion to the number of locations received each year. Two-sample Komolgorov-Smirnov tests (Systat software v. 13.1) determined whether the distribution of used and available habitats for each colony were similar and Mann-Whitney *U* tests determined whether there was a shift in the centre of the groups.

At sites except Kent Island (where the MK5540 model was exclusively used in 2013 and 2014), annual comparisons of spatial distribution and habitat use were confounded by use of different GLS models across years. Spatial and habitat data were therefore pooled across years.

## Results

**Fig 2:**
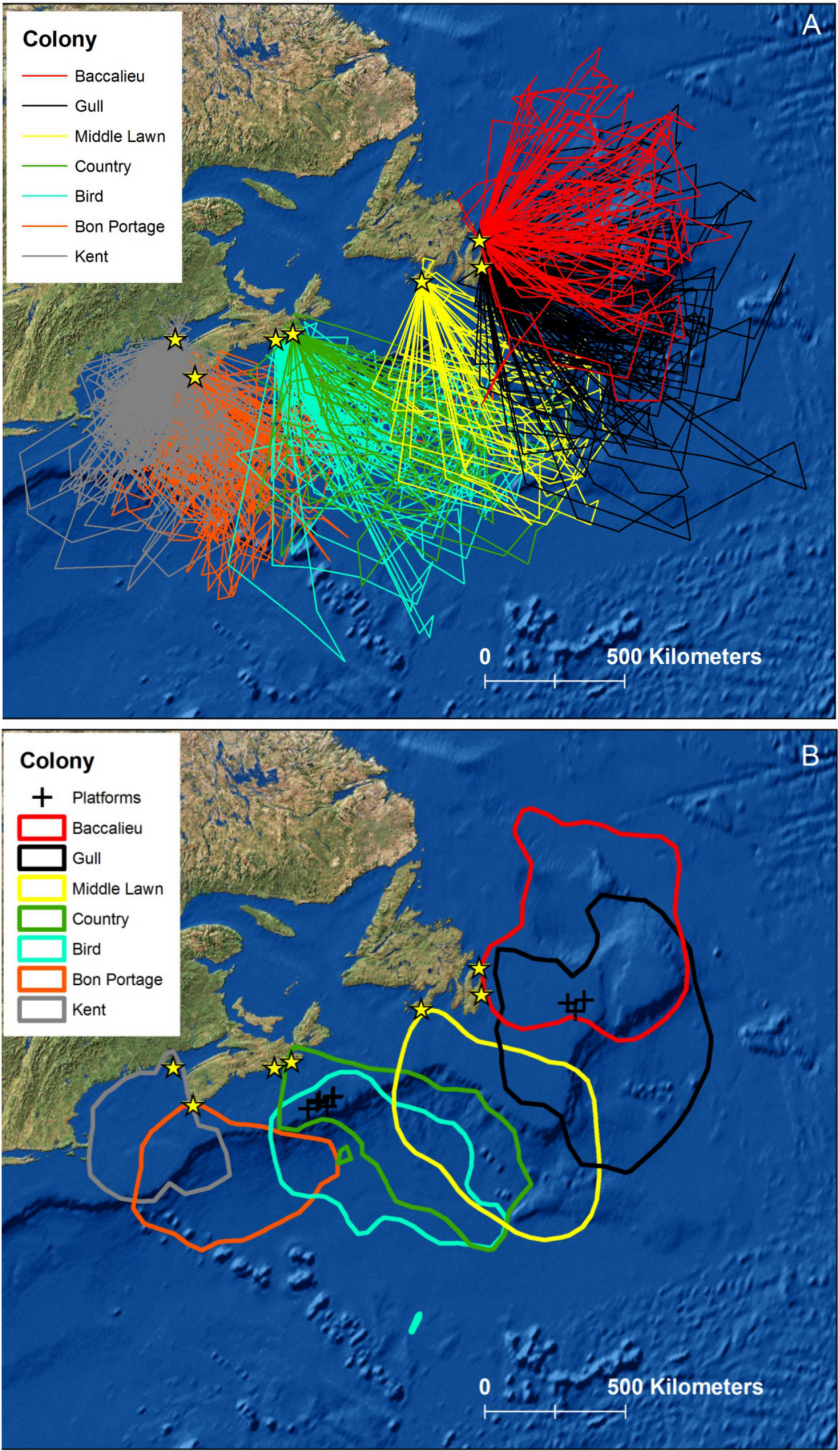
Colony foraging areas. Foraging tracks (A) and core foraging areas (B) of n = 131 Leach’s storm-petrels tracked from 7 eastern Canadian breeding colonies during the incubation period, 2013 and 2014.

The overall recovery rate of GLS from storm-petrels breeding in 7 eastern Canadian colonies in 2013 and 2014 was 74% (140 of 189; Table 1). Excluding site-year combinations for which logistical constraints limited recapture efforts (Baccalieu Island in 2013 and Middle Lawn Island in 2014) and where mammalian predators lowered breeding success (Country Island in 2013), recapture rate was higher (83%, 116 of 140; Table 1). Incorporating the latter site-year exclusions, recapture rates of sutured (79 of 97) and non-sutured (37 of 43) GLS were similar (*χ*_1_^2^ = 0.44, *p* > 0.50). Data were obtained from 95% (133 of 140) of birds recaptured; of the remainder, two had lost their GLS, four GLS failed and for a single bird the light sensor was apparently heavily shaded throughout deployment, and no useful locations were obtained. Foraging movements of 131 storm-petrels were consequently obtained (Fig 2A and Table 1); two individuals from Bon Portage Island were tracked in both 2013 and 2014. After filtering, this dataset contained 2,570 locations (S1 Dataset) collected during 405 foraging trips (S2 Dataset). While previously reported [3], our inclusion of data from Country and Bon Portage Islands in 2013 augmented spatial and colony comparisons.

Leach’s storm-petrels were highly pelagic during incubation in 2013 and 2014, in most cases travelling from coastal colonies across respective continental shelves to forage in deep oceanic waters over or beyond continental slopes (Fig 2A). Birds from Baccalieu Island concentrated over the northern Grand Bank and Flemish Cap, the Orphan Basin, and into the western Labrador Sea. Birds from Gull Island foraged south of this, over the Grand Bank and Flemish Cap, with most moving into the Newfoundland Basin. Those from Middle Lawn Island foraged in the vicinity of the Laurentian fan and along the southern and southwestern slopes of the Grand Bank. Storm-petrels from Country and Bird Islands travelled across the Scotian Shelf, the former heading southeast of the colony, again to the Laurentian fan and southwestern slopes of the Grand Bank, while those from Bird Island were concentrated more westward, in pelagic waters off the central Scotian Shelf. Birds from Bon Portage Island foraged south of the colony in pelagic waters east of George’s Bank. Birds from Kent Island, Bay of Fundy, contrasted markedly with the pattern at all other colonies, and instead foraged mainly over relatively shallow neritic waters within the Gulf of Maine and over George’s Bank (Fig 2A).

**Fig 3:**
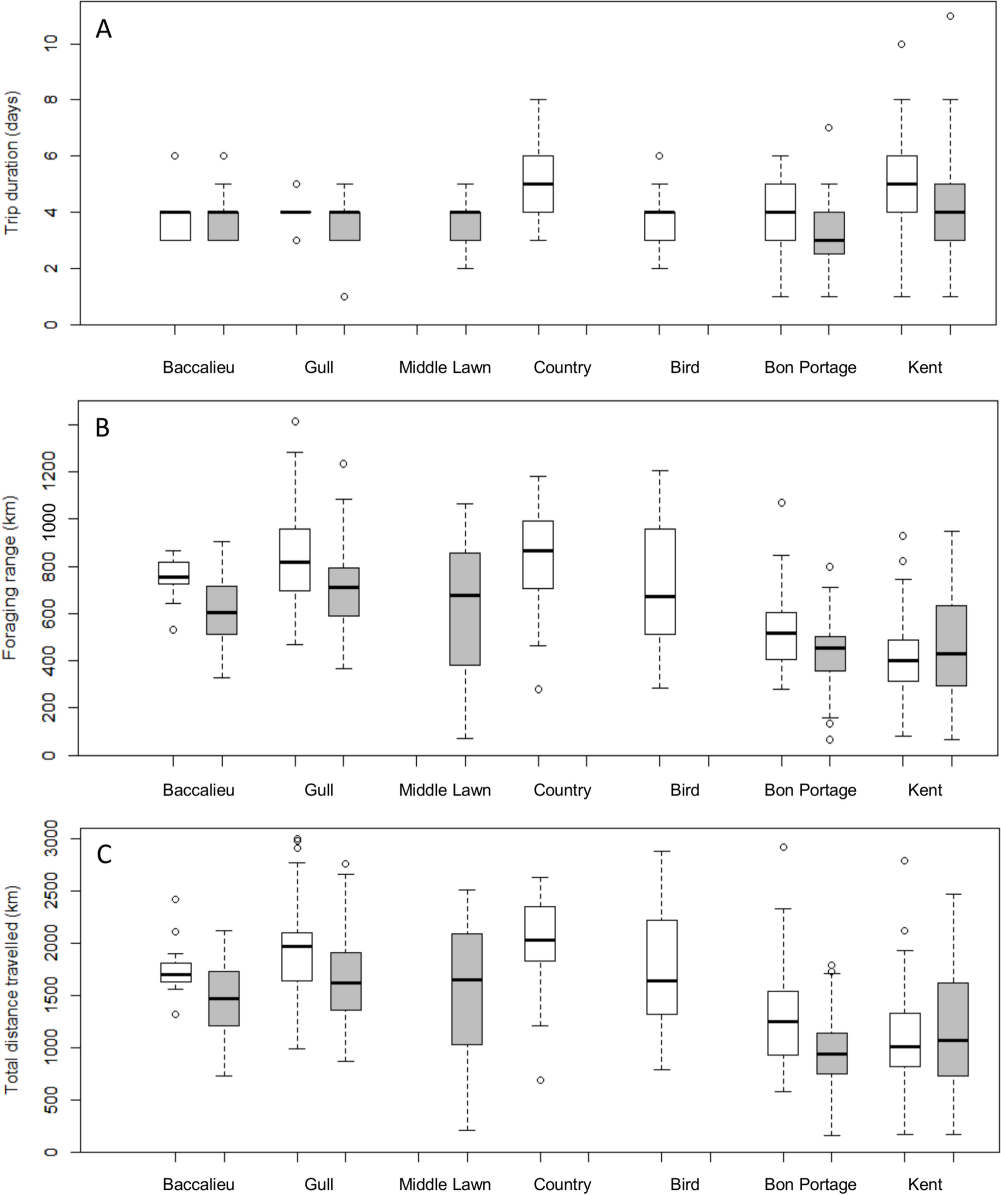
Incubation foraging trip characteristics. (A) Trip duration, (B) foraging range, and (C) total distance travelled by Leach’s storm-petrels in 2013 (white) and 2014 (grey). Box plots show the median and 25th and 75th percentiles, whiskers indicate values within 1.5 times the interquartile range, and circles represent outliers.

Despite varying in size from ~1,000 to ~ 2 million breeding pairs (Table 1), foraging trip characteristics and the size of areas used by storm-petrels at sea were independent of the log of colony size (*R*^2^ ≤ 0.17, *p* ≥ 0.36 for all metrics). Overall, foraging trip duration during incubation averaged 4.0 ± 1.4 days (n = 425; range 1–11 days), birds ranged 580 ± 243 km (n = 405; range 66–1,412 km) from their colonies and covered total cumulative distances that averaged 1,410 ± 592 km (n = 405; range 159–3,490 km; Table 2 and Fig 3). Trip durations differed among colonies in 2013 (*F*_5,58_ = 6.50, *p* < 0.001), but not in 2014 (*F*_4,64_ = 1.76, *p* = 0.15). In 2013, birds from Kent and Country Islands spent more time at sea than birds from other colonies and, at Kent Island in particular, foraging trip duration tended to be more variable (Table 2 and Fig 3A). Storm-petrel foraging ranges and cumulative travel distances differed among colonies in both 2013 (*F*_5,58_ = 15.50, *p* < 0.001 and *F*_5,58_ = 13.56, *p* < 0.001, respectively) and 2014 (*F*_4,64_ = 9.66, *p* < 0.001 and *F*_4,64_ = 9.31, *p* < 0.001, respectively). Although patterns were slightly more pronounced in 2013, birds from the more southerly sites (Bon Portage and Kent Islands) tended to forage closer to their colonies (Fig 3B) and travelled shorter distances overall (Fig 3C).

**Table 2:** Characteristics of Leach’s storm-petrel foraging trips during the incubation period, 2013 and 2014. Values are means ± standard deviations (sd) of average values for individual birds. Colonies are listed by latitude from north to south.

Year	Colony	N birds	Trip duration (d)		Foraging range (km)		Cumulative distance (km)	
			Mean ± sd	N trips	Mean ± sd	N trips	Mean ± sd	N trips
2013	Baccalieu Is., NL	6	3.9 ± 0.2	14	754 ± 73	14	1757 ± 154	14
	Gull Is., NL	7	3.9 ± 0.3	28	832 ± 156	28	1954 ± 327	28
	Country Is., NS	9	5.1 ± 0.6	18	833 ± 163	18	2062 ± 416	18
	Bird Is., NS	11	4.0 ± 0.6	36	763 ± 245	33	1793 ± 517	33
	Bon Portage Is., NS	14	4.1 ± 0.6	38	510 ± 110	36	1261 ± 268	36
	Kent Is., NB	17	5.0 ± 0.9	71	412 ± 130	70	1089 ± 328	70
2014	Baccalieu Is., NL	13	4.0 ± 0.5	36	601 ± 105	36	1457 ± 186	36
	Gull Is., NL	16	3.7 ± 0.4	52	698 ± 123	50	1640 ± 327	50
	Middle Lawn Is., NL	9	3.8 ± 0.5	31	637 ± 235	30	1531 ± 534	30
	Bon Portage Is., NS	16	3.5 ± 1.1	39	400 ± 129	37	919 ± 310	37
	Kent Is., NB	15	4.4 ± 1.0	62	482 ± 156	53	1217 ± 401	53

In 2013, Bayesian state space modelling [3] produced mean foraging ranges (983 ± 249 and 587 ± 149 km) and cumulative travel distances (2,117 ± 541 and 1,371 ± 379 km) for Country and Bon Portage Islands, respectively, that exceeded our threshold derived estimates by an average of just 11 ± 6.9% (~50–150 km; Table 2). In addition, estimated foraging trip characteristics obtained using high-precision GPS at Gull Island in 2016 (trip duration: 3.6 ± 0.6 days, n = 11; foraging range: 657 ± 113 km, n = 19; cumulative distance: 1,544 ± 187 km, n = 11) were similar to those estimated from GLS at this site, particularly in 2014 (Table 2).

**Fig 4:**
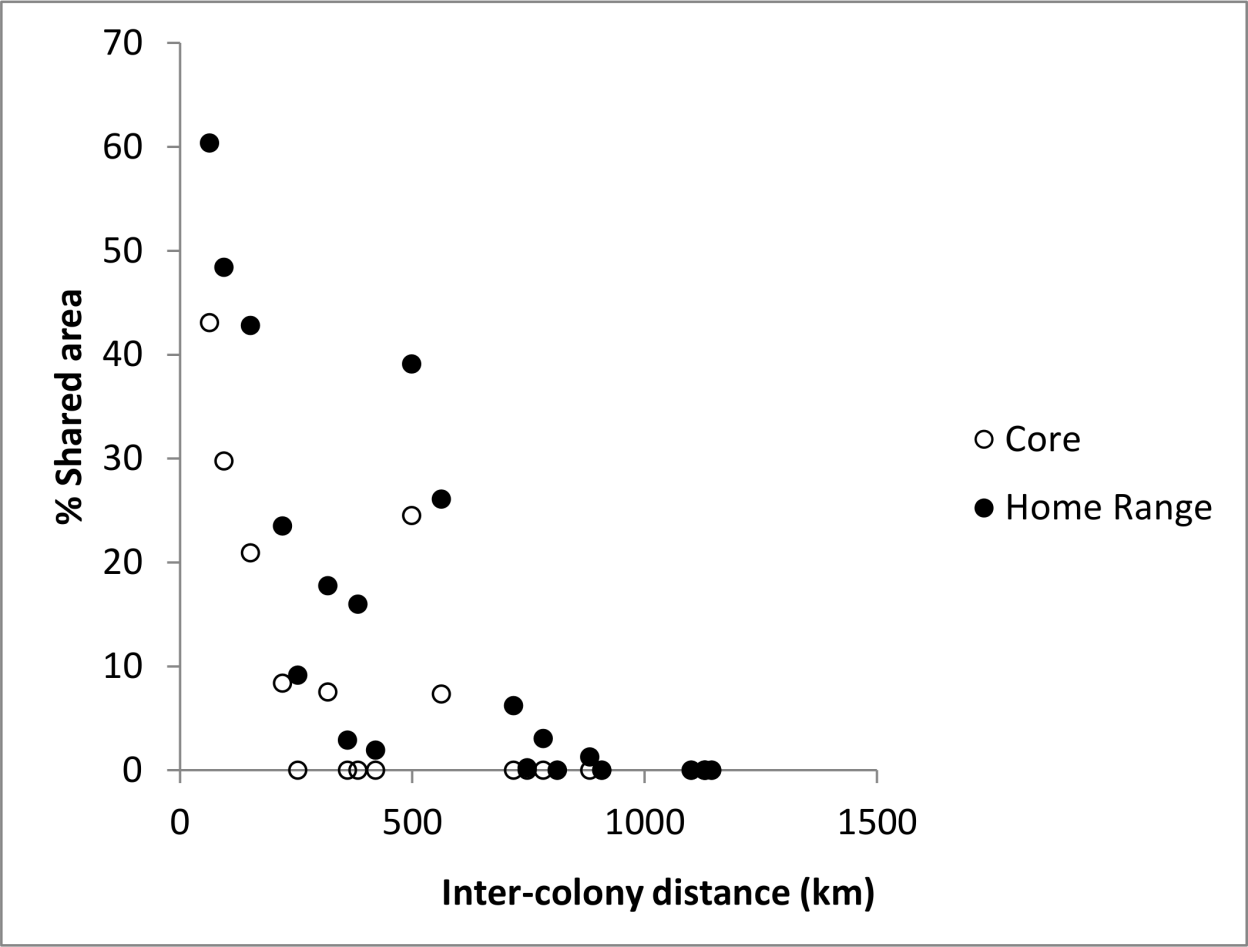
Extent of spatial overlap among colonies. Influence of inter-colony distance (km) on the degree of overlap in core and home range areas for 7 colonies of Leach’s Storm-petrels breeding in eastern Canada.

Smoothing parameters (*h* values) for the UDs averaged 63.8 ± 19.8 km and colony core areas were bounded, on average, by the 69 ± 1.3% isopleth (Fig 2B). Despite being capable of covering vast distances during the incubation period, birds tended to forage in deep water areas adjacent to their breeding colonies (Fig 2). Owing to this strategy, the extent of overlap among colonies was low overall and negatively related to the distance between them (Fig 4). Core areas overlapped only for colonies separated by ≤ 560 km, while home range overlap was evident for colonies ≤ 880 km apart (Fig 4). Core areas did not overlap at all for 4 of 11 colony-pairs separated by ≤ 560 km, and the average for those showing some overlap was just 20% (range 7–43%; Table 3). For 16 colony-pairs separated by ≤ 880 km, home ranges overlapped by an average of 19% (range 0–60%; Table 3). Maximum pairwise overlap (43% for core and 60% for home range areas) occurred for Country and Bird Islands which are separated by just 64 km (Table 3). At Kent Island, foraging areas during incubation in 2013 and 2014 were highly similar; 66% of home range and 67% of core areas were shared between years.

**Table 3:** Percentage of core and home range (95% UD) areas shared by colonies of Leach’s storm-petrels during incubation. Colonies are listed by latitude from north to south.

	**% Area Shared**						
	**Core Area**						
	**Baccalieu**	**Gull**	**Middle Lawn**	**Country**	**Bird**	**Bon Portage**	**Kent**
Baccalieu	-						
Gull	29.8	-					
Middle Lawn	0.0	8.4	-				
Country	0.0	0.0	24.5	-			
Bird	0.0	0.0	7.3	43.1	-		
Bon Portage	0.0	0.0	0.0	0.0	7.5	-	
Kent	0.0	0.0	0.0	0.0	0.0	20.9	-
	**Home Range Area (95% UD)**						
	**Baccalieu**	**Gull**	**Middle Lawn**	**Country**	**Bird**	**Bon Portage**	**Kent**
Baccalieu	-						
Gull	48.4	-					
Middle Lawn	9.1	23.5	-				
Country	0.2	6.2	39.1	-			
Bird	0.0	3.0	26.1	60.4	-		
Bon Portage	0.0	0.0	1.3	16.0	17.8	-	
Kent	0.0	0.0	0.0	1.9	2.9	42.8	-

**Fig 5:**
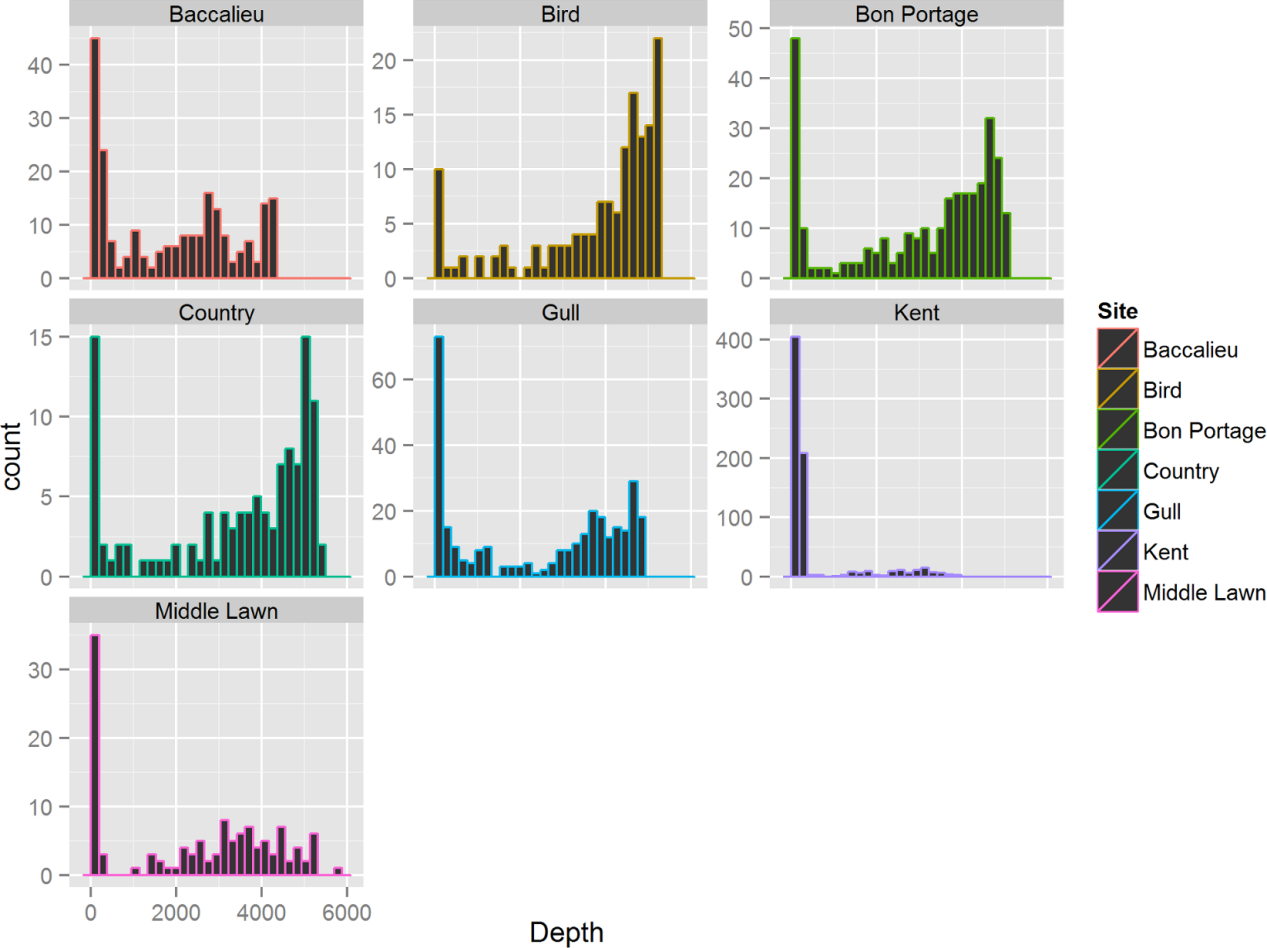
Ocean depth within core areas. Distribution of depths (m) recorded within core areas of Leach’s storm-petrels from 7 eastern Canadian breeding colonies.

The distribution of habitat characteristics (bathymetry, SST, and chl *a*) was significantly different in areas used by (i.e., core area) and available to (i.e., within the mean maximum foraging range) Leach’s storm-petrels (Two-sample Komolgorov-Smirnov tests, *p* < 0.01 for all variable by colony comparisons). Throughout incubation, depths within colony core areas exceeded those in available habitats (Mann-Whitney *U* tests, *p* < 0.001 for all colonies), and for all colonies except Kent Island were associated with offshore pelagic habitats (median depth > 1,950 m; Fig 5) with average chl *a* concentrations ≤ 0.6 mg/m^3^ (Table 4). SSTs within colony foraging zones varied widely, spanning > 11°C. Birds from Baccalieu and Gull Islands foraged in cold offshore waters influenced by the Labrador Current over and beyond the continental slope of the Grand Bank and Orphan Basin. SSTs within these areas were below those in the available habitat (Mann-Whitney *U* tests, *p* < 0.001 for both colonies) and averaged < 13°C. Birds from Nova Scotia colonies, in contrast, foraged either off the Scotian Shelf or southwest of the Grand Bank in waters influenced by the Gulf Stream, and experienced SSTs exceeding those in the available habitat (Mann-Whitney *U* tests, *p* < 0.01 for all colonies), and averaging > 21°C (Table 4). Birds from Kent Island mainly used more shallow neritic waters (median depth = 181 m) within the Gulf of Maine and George’s Bank that had moderate SST (15.7 ± 1.4°C) and the highest recorded chl *a* concentrations among the colonies (1.1 ± 0.3 mg/m^3^).

**Table 4:** Habitat characteristics within core areas of Leach’s storm-petrels from eastern Canadian breeding colonies during incubation, 2013 and 2014. Colonies are listed by latitude from north to south. SST and Chl *a* values are means ± SD of average values for individual birds. Depths were not normally distributed and are listed as median values.

Colony	N bird records	Depth (m)	SST (°C)	Chl *a* (mg/m^3^)
Baccalieu Is., NL	19	1946	11.1 ± 1.7	0.6 ± 0.2
Gull Is., NL	23	3106	13.0 ± 2.4	0.4 ± 0.1
Middle Lawn Is., NL	9	2919	16.8 ± 2.7	0.4 ± 0.1
Country Is., NS	9	3994	21.8 ± 1.7	0.3 ± 0.0
Bird Is., NS	11	4456	22.7 ± 1.8	0.2 ± 0.1
Bon Portage Is., NS	30	3675	21.4 ± 2.1	0.5 ± 0.4
Kent Is., NB	32	181	15.7 ± 1.4	1.1 ± 0.3

Oil and gas production platforms intersected the core area of incubating birds from Baccalieu, Gull, Country, and Bird Islands (Fig 2B), and the peripheral portion of the range (95% UD) of birds from Bon Portage Island. There are currently no platforms operating within areas used by birds from Middle Lawn and Kent Islands during the incubation period.

## Discussion

Across a network of colonies, that almost spanned the Northwest Atlantic breeding range, Leach’s storm-petrels exhibited highly pelagic foraging behavior during the incubation period. On a typical 4-day foraging trip, birds from most colonies ranged > 500 km from coastal breeding sites crossing continental shelves to forage in open oceanic waters over and beyond continental slopes, travelling, on average, > 1,400 km per round trip. Offshore foraging habitats were characterized by waters with median depth > 1,950 m and chl *a* concentrations ≤ 0.6 mg/m^3^. Average SSTs varied markedly among colonies (11–22° C) coincident with the water mass predominating within the foraging areas. At Kent Island, close to the southern limit of the species’ breeding range in eastern North America, results contrasted with this general pattern, as birds foraged mainly in more shallow neritic waters (median = 181 m) within the Gulf of Maine. Birds travelled vast distances during incubation but they tended to forage in areas adjacent to their colonies. Owing to this, the extent of spatial overlap among colonies was low (~20%) and negatively related to the distance between them.

With an overall recovery rate ≥ 74% and previous reports of no effect on body mass, hatching or fledging success for Leach’s storm-petrels [3], suturing proved an effective attachment technique for studying foraging behaviour during the incubation period. However, lower chick growth for birds carrying sutured GLS [3], suggests that the potential for impacts during more energetically taxing (or more sensitive) breeding phases, or during deployments of extended duration, should be recognized.

While GLS positions derived using the threshold method typically have poor spatial resolution, two lines of evidence suggest this method is appropriate for depicting the foraging movements of incubating storm-petrels in our region. First, our estimates of foraging parameters were similar to those derived using Bayesian techniques which control for error in location estimates [3]. Estimates of mean foraging ranges and cumulative travel distances derived from state space models for Country and Bon Portage Islands in 2013 [3], exceeded those presented here by an average of just 11 ± 6.9%. In addition, while we could expect foraging metrics for Leach’s storm-petrels to vary among years [3], estimates of travel distances obtained using high precision GPS at Gull Island in 2016 were similar to those obtained using GLS at this site, particularly in 2014, thus further validating the use of GLS to depict the species’ foraging distribution during the incubation period. While the latter comparison was drawn to demonstrate the suitability of GLS in this instance, we strongly caution against inter-colony or inter-annual comparisons of data derived from devices varying in spatial precision, because errors inherent in GLS, for example, can result in inflation of foraging ranges [28].

Avian distribution at sea is linked strongly to the distribution of prey, which is driven by biophysical ocean dynamics [38]. Leach’s storm-petrels covered substantial distances while foraging during the incubation period, moving into open oceanic waters where they would have had access to abundant mesopelagic prey [39]. Both in Newfoundland and Nova Scotia, Leach’s storm-petrels rely heavily on mesopelagic lantern-fishes while raising chicks [10,11,40]. The bird’s distribution at sea suggests that these deepwater fish are also likely important components of the diet during incubation. Myctophids are globally abundant, energy-rich [41] fish found predominantly in open oceanic environments [42,43]. They occur at depth by day but undergo diel vertical migration, feeding within the epipelagic zone at night when they would become available to surface-feeding storm-petrels. Recent studies in Newfoundland indicated that the glacier lantern-fish, *Benthosema glaciale*, an important prey of Leach’s storm-petrel at both Baccalieu and Gull Islands [10, 11], is abundant (average of 6 fish m^-2^ and biomass of 9.3 g m^-2^) within the western Labrador Sea [44]. Furthermore, unlike the patchy nature of many prey species targeted by seabirds, glacier lantern-fish have a nearly continuous distribution, from the continental slope of the Grand Bank into the central portion of the Labrador Sea [44]. The apparent continuous distribution of abundant, high quality prey coupled with the birds’ low wing-loading, and hence low cost of flight [45], seem to at least partially explain the observed distributional patterns.

Operating within the constraints of central place foraging, varied space use at sea is one mechanism through which neighbouring seabird colonies can reduce intra-specific competition for limited prey during the breeding season [46]. Colony-specific foraging areas have been reported for many species during the breeding season, including shy *Thalassarche cauta* [47], and black-browed albatrosses [48,49], Cape gannets *Morus capensis* [46], macaroni penguins *Eudyptes chrysolophus* [50], and lesser black-backed gulls *Larus fuscus* [51]. There is compelling evidence of segregation of foraging areas along colony lines in a recent study in which northern gannets *M*. *bassanus* from 12 colonies were tracked simultaneously [52]. Foraging areas of the gannets overlapped very little at sea, even among colonies situated close together, and both the size of foraging areas and foraging trip metrics were strongly dependent on colony size. Modelling the observed patterns, Wakefield et al. [52] demonstrated that patterns in gannet foraging were determined through density-dependent competition. In this study of Leach’s storm-petrels, where colony size had no influence on the size of the areas used at-sea or on other foraging trip metrics, and where extent of overlap among colonies was negatively related to inter-colony distance, there was little indication that intra-specific competition influenced observed spatial patterns. Rather, availability of pelagic habitat areas, at and beyond shelf edges, where birds have access to apparently widely available and abundant mesopelagic resources [44], resulted in little spatial overlap among colonies.

Spatially distinct and temporally consistent foraging areas of Leach’s storm-petrels carry both ecological and conservation implications. Largely separate foraging areas could result in colonies sampling separate prey fields during the breeding season, to the extent that prey are either non-migratory or that different prey species associate with different water masses. Myctophids, for example, occur globally, but the species composition varies regionally [43]. Mercury concentrations in mesopelagic fish such as the myctophids are 4-fold higher than in epipelagic fish occupying the same trophic level [53]. Because mercury concentrations in Leach’s storm-petrel eggs and other tissues are relatively high in Atlantic Canada [20–23,54], the influence of diet composition on contaminant loads needs to be further investigated. Differential exposure to mercury has been found in other seabirds wintering in different areas of the Northwest Atlantic [55].

Spatially discrete risks for storm-petrels, as well as other seabirds, are imposed by activities surrounding the extraction of oil and natural gas off the east coast of Canada [24–26,56–60]. The main risks for storm-petrels include attraction to lights and flares at platforms and related structures and hydrocarbon contamination from operational discharges and spills [26,59,60]. Mortality from the former results from collisions and strikes as well as incineration in flares, but a lack of information on avian attraction and interaction with platforms precludes assessment of its likely significance [57]. We demonstrate that the foraging areas of incubating birds from Baccalieu and Gull Islands in Newfoundland and Country, Bird, and Bon Portage Islands in Nova Scotia overlap with current oil and gas production areas (Fig 2). Populations at Baccalieu, Gull, and Country Islands are declining, while the status of populations at Bird and Bon Portage Islands are unknown (Table 1). Breeders from Middle Lawn and Kent Islands forage largely outside current production areas (Fig 2B). The Middle Lawn Island population has declined over the past couple of decades as a result of gull predation [15], and the population trend at Kent Island is unknown. As offshore hydrocarbon exploration, development, and production operations increase in Atlantic Canada [61], risks from these facilities will presumably increase. To assess and address environmental effects, there is an urgent need for quantitative information on avian attraction and interaction with offshore platforms off Canada’s east coast [26,57–60].

This study has increased knowledge of broad-scale spatial distribution of incubating Leach’s storm-petrels throughout their breeding range in the Northwest Atlantic. Further fine-scale spatial (i.e., GPS-quality) information, however, is required to better assess interactions with and potential impacts of spatially discrete risks, both on the breeding and the wintering grounds (the latter remain largely unknown, but see [4]). Future efforts should prioritize modelling to incorporate information on relative predation risk at colonies, spatially discrete risks at-sea (year-round), both lethal and sub-lethal effects of environmental contaminants, as well as potential effects of climate and marine ecosystem change to better understand the likely cumulative factors driving declining population trends of Leach’s storm-petrels in Atlantic Canada.

## Supporting information

S1 Dataset Processed and filtered GLS positions according to individual and colony. (XLSX)

S2 Dataset Incubation foraging trip metrics according to individual and colony. (XLSX)
